# Self-regulation of the inflammatory response by peroxisome proliferator-activated receptors

**DOI:** 10.1007/s00011-019-01231-1

**Published:** 2019-03-29

**Authors:** Jan Korbecki, Rafał Bobiński, Mieczysław Dutka

**Affiliations:** 1 Department of Molecular Biology, School of Medicine in Katowice, Medical University of Silesia, Medyków 18 Str., 40-752 Katowice, Poland; 2 Department of Biochemistry and Molecular Biology, Faculty of Health Sciences, University of Bielsko-Biala, Willowa 2 Str., 43-309 Bielsko-Biała, Poland

**Keywords:** Inflammation, Peroxisome proliferator-activated receptor, Cyclooxygenase-2, NF-κB, Signaling pathway

## Abstract

The peroxisome proliferator-activated receptor (PPAR) family includes three transcription factors: PPARα, PPARβ/δ, and PPARγ. PPAR are nuclear receptors activated by oxidised and nitrated fatty acid derivatives as well as by cyclopentenone prostaglandins (PGA_2_ and 15d-PGJ_2_) during the inflammatory response. This results in the modulation of the pro-inflammatory response, preventing it from being excessively activated. Other activators of these receptors are nonsteroidal anti-inflammatory drug (NSAID) and fatty acids, especially polyunsaturated fatty acid (PUFA) (arachidonic acid, ALA, EPA, and DHA). The main function of PPAR during the inflammatory reaction is to promote the inactivation of NF-κB. Possible mechanisms of inactivation include direct binding and thus inactivation of p65 NF-κB or ubiquitination leading to proteolytic degradation of p65 NF-κB. PPAR also exert indirect effects on NF-κB. They promote the expression of antioxidant enzymes, such as catalase, superoxide dismutase, or heme oxygenase-1, resulting in a reduction in the concentration of reactive oxygen species (ROS), i.e., secondary transmitters in inflammatory reactions. PPAR also cause an increase in the expression of IκBα, SIRT1, and PTEN, which interferes with the activation and function of NF-κB in inflammatory reactions.

## Background

The prostaglandin synthesis pathway is an important element of inflammatory responses. The pathway is induced by cytokines [1, 2], LPS [3, 4], or xenobiotics, including metal compounds [5–7] and fluoride [8–10]. At the beginning of the pathway, PLA_2_ releases arachidonic acid (AA) from the cell membranes [11]. Next, AA is enzymatically converted, at first by lipoxygenases (LOX) or cytochromes P450 (CYP), or by the best known COX, into a large group of compounds called eicosanoids [12, 13]. Like any biochemical response, increasing synthesis of various eicosanoids in the COX pathway is subject to strict regulation. There are numerous regulatory mechanisms in this pathway which cause both an increase and decrease in synthesis and in activity of particular eicosanoids [14, 15]. Regulation relating to modification of activity of prostaglandins and thromboxanes receptors is an example of the above [16]. The activity of COX-2 is also enhanced by the product of its pathway, i.e., by PGE_2_ in an autocrine manner [17, 18]. Activation of prostaglandin E_2_ receptors (EPs) causes an increase in the level of cAMP and activation of the cAMP response element-binding protein (CREB) which leads to increased COX-2 expression [19, 20]. The increase in COX-2 expression may also depend on the activation of mitogen-activated protein kinase (MAPK) cascades [21] and phosphoinositide 3-kinase (PI3K) [18, 22]. Apart from the effect of individual products of the COX pathway, LOX products also increase COX-2 expression [23]. Despite this, AA, regardless of COX, LOX, and CYP activity, is itself capable of causing oxidative stress and, in particular, of activating NADPH oxidase [24, 25]. This results in an increase in the level of reactive oxygen species (ROS) and activation of c-Jun N-terminal kinase (JNK) MAPK and NF-κB, which increases COX-2 expression.

**Fig. 1:**
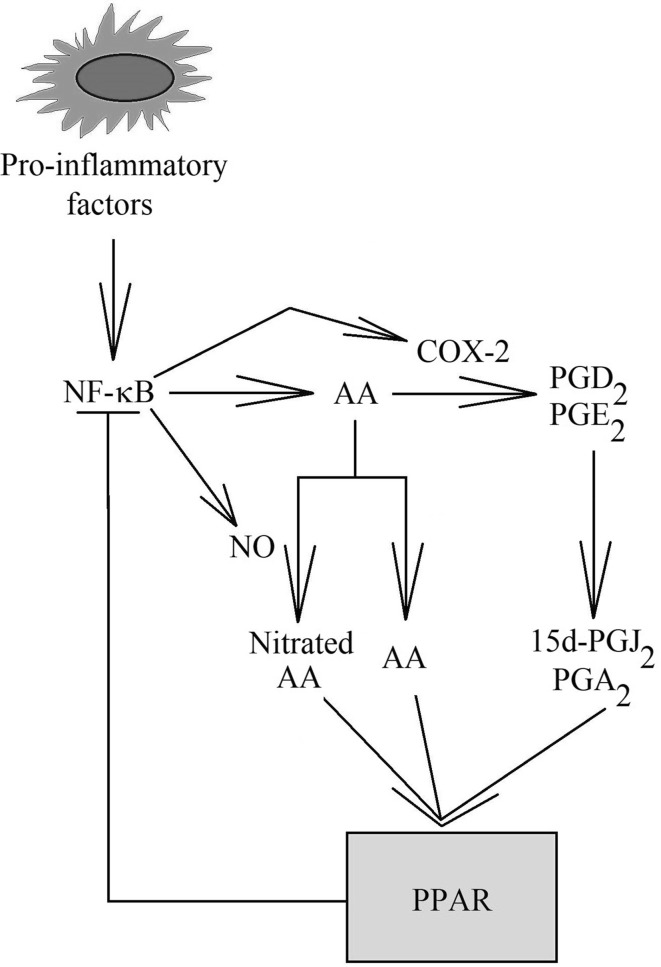
Self**-**regulation of NF-κB activity and COX-2 expression. In inflammatory reactions, NF-κB is activated and, partly as a result of this, an increase in expression and activity of enzymes of the prostaglandin synthesis pathway takes place. Released AA is converted into PGD_2_ or PGE_2_. In inflammatory reactions, the production and concentration of NO also increase. With time, all of the compounds react together or undergo further non-enzymatic transformation. AA in reaction with NO is subject to nitration. PGD_2_ and PGE_2_ convert to 15d-PGJ_2_ and PGA_2,_ respectively. Compounds with anti-inflammatory properties are formed, which activate PPARα and PPARγ. Activated PPARα and PPARγ inhibit the activity of NF-κB, which leads to inhibition of inflammatory reactions by the products of these reactions

Nonetheless, self-regulation of the COX pathway does not consist solely of positive feedback. The pathway also involves mechanisms which inhibit a too strong inflammatory response. In the center of the COX auto-inhibition pathway, peroxisome proliferator-activated receptors (PPAR) are found (Fig. 1). Activation of inflammatory responses by LPS or other pro-inflammatory particles causes an increase in the expression of PPARα and a decrease in the expression of PPARγ [26–29]. Blocking the mechanism with a knock-out gene for PPARα causes an increase in the intensity of inflammatory responses to IL-1 or LPS [28, 30].

## Ligands of peroxisome proliferator-activated receptors

PPAR are nuclear receptors and transcription factors. They include PPARα, PPARβ/δ, and PPARγ. These transcription factors control genes responsible for the oxidation of lipids [31, 32]. PPARα is a transcription factor that increases gene expression of acyl-CoA oxidase and carnitine palmitoyltransferase I, i.e., enzymes involved in β-oxidation. PPARγ increases adiponectin concentration and expression of transporters GLUT1 and GLUT4. PPARβ/δ increases the expression of pyruvate dehydrogenase kinase-4 and carnitine palmitoyltransferase 1A, which increases the intensity of fatty acid oxidation.

All of PPAR have a similar structure—ligand-binding domain (LBD). LBD is in the shape of the letter Y and is of polar character [33–36]. In the first arm, hydrophilic amino acid residue can be found, which is responsible for ligand binding. This part also contains helix12, which is stabilized during ligand binding and PPAR activation. The remaining two arms of the ligand-binding domain consist of hydrophobic amino acid residue with far less hydrophilic residue. These two parts of LBD are responsible for the specificity of ligand binding when activating PPAR.

This LBD structure enables the activation of PPAR by polar structure ligands, and, in particular, by a fatty acids and derivatives of fatty acids (Table 1). Nonetheless, not all fatty acids equally activate PPAR. The arms with hydrophobic residue in LBD stabilize ligand [33]. Therefore, only fatty acids with 14 and more carbon atoms are capable of activating PPAR [33, 37, 38]. However, saturated fatty acids with 20 and more carbon atoms do not fit in the LBD and thus are not activators of PPAR. Double bonds also play an important role in the structure of a fatty acid as a potential ligand. Monounsaturated fatty acids in *cis* configuration have a favorable conformation to better match LBD than saturated fatty acids and fatty acids in *trans* configuration of the same length. Fatty acids in *trans* configuration have similar conformation as unsaturated fatty acids [33]. Long-chain polyunsaturated fatty acid (PUFA) is also ligands for PPAR [33, 37, 39, 40]. For example, AA connects with LBD at a concentration of IC_50_ equal to 1.2 ± 0.2 μM and 1.6 ± 0.2 μM for PPARα and PPARγ, respectively [33, 41]. Eicosapentaenoic acid (EPA) and docosahexaenoic acid (DHA) have similar properties. However, at a much higher concentration, 100 μM, inhibition of PPARα activity by AA, EPA, and DHA takes place [38].

**Table 1:** Examples of PPAR agonist

Agonist	PPARα	PPARβ/δ	PPARγ	Bibliography
Saturated fatty acids	Palmitic acid, stearic acid	Palmitic acid, stearic acid	–	[33, 37]
Monounsaturated fatty acids	Palmitoleic acid, oleic acid	Palmitoleic acid, oleic acid	Palmitoleic acid, oleic acid	[33]
Polyunsaturated fatty acid	γ-LA, AA, EPA, DHA	γ-LA, AA, EPA, DHA	γ-LA, AA, EPA, DHA	[33, 37, 40]
Arachidonic acid metabolites	LTB_2_, 8(*S*)-HETE, EET, 20-COOH-AA	8(*S*)-HETE, 15-HETE	8(*S*)-HETE, 15-HETE, EET, 20-COOH-AA, PGA_2_, 15d-PGJ_2_	[37, 40, 44, 46–49, 51]
Other derivatives of fatty acids	Nitrated derivatives of unsaturated fatty acids	–	Nitrated derivatives of unsaturated fatty acids	[65, 66]
Synthetic agonist	Wy 14,643 Ciprofibrate, clofibrate, bezafibrate, ETYA	Bezafibrate, ETYA	Ciprofibrate, clofibrate, BRL 49653, NSAID (diclofenac, flufenamic acid, flurbiprofen, indomethacin, NS-398)	[37, 40, 109–111]

15d-PGJ_2_: 15-deoxy-Δ^12,14^-prostaglandin J_2_; AA: arachidonic acid; γ-LA: γ-linolenic acid; DHA: docosahexaenoic acid; EET: epoxyeicosatrienoic acid; EPA: eicosapentaenoic acid; ETYA: 5,8,11,14-eicosatetraynoic acid; HETE: hydroxyeicosatetraenoic acid; LTB_2_: leukotriene B_4_; NSAID: nonsteroidal anti-inflammatory drug; PGA_2_: prostaglandin A_2_

Hydrophilic residue is also present in LBD, which enables activation of PPAR by derivatives of fatty acids arising from enzymatic and non-enzymatic oxidation [35, 42]. Owing to self-regulation of inflammatory responses, AA metabolites fulfill special roles as activators. An example of the above is the products of the 5-LOX pathway, 5(*S*)-hydroxyeicosatetraenoic acid (5(*S*)-HETE), and leukotriene B_4_ (LTB_4_), which also activate PPARα [37, 40, 43]. Nonetheless, this effect is less significant compared to the impact of free AA—fatty acids from which they arise. Other important activators of PPAR are 8(*S*)-HETE and hydroperoxyeicosatetraenoic acid (8*S*-HPETE)—products of murine 8-LOX [37, 40, 44, 45]. Yet, another important activator of PPARγ and PPARβ/δ is the 15-LOX product: 15-HETE [46, 47]. Among natural activators of PPARα and PPARγ are AA metabolites, products of many CYP isoforms with anti-inflammatory properties are also found. They include: 5,6-epoxyeicosatrienoic acid (EET), 8,9-EET, 11,12-EET, 14,15-EET, and 20-HETE (with its metabolite 20-COOH-AA) [13, 48–51].

**Fig. 2:**
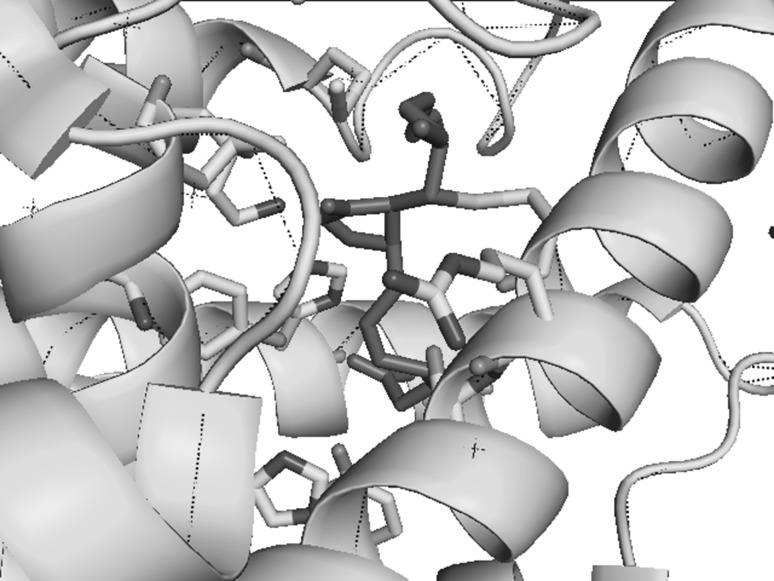
15d-PGJ_2_ as an agonist of PPARγ. Modeled (as purple) 15d-PGJ_2_ in LBD PPARγ. Amino acid residues interacting with this ligand and covalently bound Cys^285^ are shown. PDB:2zk1 [54]

Prostaglandins produced in the COX pathway have impact on activating PPARγ. Cyclopentenone prostaglandins activate PPARγ [37, 44, 52]. This group includes prostaglandin (PG)A_2_, PGC_2_, PGJ_2_, Δ^12^-PGJ_2,_ and the most thoroughly studied 15-deoxy-Δ^12,14^-prostaglandin J_2_ (15d-PGJ_2_). All prostaglandins in this group arise from pro-inflammatory prostaglandins. PGA_2_ is formed as a result of non-enzymatic dehydration of PGE_2_. Subsequently, PGA_2_ may be isomerized to PGB_2_ and PGC_2_, whereas PGD_2_ may be subject to non-enzymatic dehydration and isomerisation to PGJ_2_, Δ^12^-PGJ_2_ and, finally, to 15d-PGJ_2_. All of the cyclopentenone prostaglandins contain an electrophilic carbon atom in their cyclopentenone ring [53, 54]. Owing to this, they can be subject to Michael addition to free thiol (–SH) groups in cysteine. If such residue is present in the catalytic center or in an important domain in the function of a given protein, such enzymes and transcription factors become inactive. IκB kinase β subunit (IKKβ) and nuclear factor κB (NF-κB) are examples of such proteins [52, 55]. Cysteine is also present in LBD PPARγ in position 285 (Cys^285^), which is specifically susceptible to reactions and covalent 15d-PGJ_2_ connection (Fig. 2) [40, 54, 56]. As a result of alkylation of this residue, a change in PPARγ conformation takes place and the protein is activated [37, 39, 40, 44]. The cyclopentenone prostaglandins constitute negative feedback to inflammatory responses. During the inflammatory responses, an increase in the production of PGE_2_ and PGD_2_ is observed [57]. With time, in a non-enzymatic way, further transformation of the prostaglandins into anti-inflammatory compounds takes place in PGA_2_ and 15d-PGJ_2,_ respectively. As a consequence, the inflammatory response is reduced by transformed products of the responses [3, 58].

In the course of inflammatory reactions, an increased synthesis occurs of nitric oxide (NO) and ROS generation, among others superoxide radical (O_2_^•−^), and hydrogen peroxide (H_2_O_2_) [59, 60]. NO may react with O_2_^•−^ creating peroxynitrite (ONOO^−^). Both compounds, NO and ONOO^−^, lead to nitration of double bonds in unsaturated fatty acids [42, 61]. As a result, nitrated derivatives of fatty acids are formed. These compounds have biological properties of an anti-inflammatory nature. One of their functions is binding-free thiol (–SH) groups in cysteine [62, 63]. Owing to this, they may cause nitroalkylation of Cys^62^ p50 NF-κB and Cys^38^ p65 NF-κB, which, as a consequence, inactivates these transcription factor subunits. A nitrated derivative of AA replaces heme in COX, which impacts the intensity of inflammatory responses. This results in irreversible inhibition of the enzymes K_i_ for COX-1, equal to 1.02 μM, and K_i_ for COX-2, equal to 1.76 μM [64]. Another anti-inflammatory characteristic of nitrated derivatives of fatty acids, in very low concentrations, is activation of PPARα and PPARγ [65, 66]. In PPARγ, meanwhile, there are residues of Arg^288^ and Glu^343^ which stabilize, respectively, the nitrated derivatives of fatty acids on 10 and 12 carbon [67]. The above-mentioned residues of Arg^288^ and Glu^343^ are not conservative and hence do not occur in other PPAR. Owing to such structure, PPARγ is activated by the nitrated derivatives of unsaturated fatty acids even at a concentration of 100 nM [65, 66]. Meanwhile, concentration of PPARα deprived of such residues occurs at a concentration of 300 nM [65]. For higher concentrations of about 4 μM, nitrated derivatives of fatty acids, and of AA in particular, unbalance the assembly of the NADPH oxidase complex [68]. This causes disturbance in ROS generation and, as a result, suppresses inflammatory reactions.

## Activation of peroxisome proliferator-activated receptors

PPARγ is a transcription factor, whose activity is subject to regulation by SUMOylation and an assembly of complexes with various proteins, especially with nuclear receptor corepressor (NCoR) and silencing mediator of retinoid and thyroid hormone receptors (SMRT) [69–73]. Activation of PPARγ with ligand causes changes in SUMOylation of this protein. In particular, a decrease takes place in SUMOylation of Lys^33^ PPARγ1 in the domain of activation function 1 (AF1) [74]. This allows LBD to interact with the AF1 domain, whereas SUMOylation of the Lys^365^ in LBD PPARγ1 allows for full activation of PPARγ1 by ligand. PPARγ activation also causes suppression of proteolytic degradation of NCoR and dissociation of the complex of PPARγ with NCoR and SMRT [69, 72]. As a result, expression of genes is suppressed by these corepressors, especially of the genes dependent on NF-κB and AP-1, such as *PTGS2* and *NOS2* [75–77].

A similar mechanism is present in PPARα. The transcription factor in its inactive form is subject to SUMOylation and it is also in complex with NCoR [78, 79]. The process of SUMOylation is governed by enzymes Ubc9 and PIASy, which modify the Lys^185^ PPARα. Activation of PPARα by ligand leads to a decrease in SUMOylation and, as a consequence, to release of NCoR. NCoR is a corepressor which connects exclusively to SUMOylated receptors. Dissociation of the complex of PPARα with NCoR leads to activation of both proteins; PPARα increases expression of particular genes and NCoR is a corepressor suppressing the expression of other genes, including *PTGS2* and *NOS2* [77].

## Changes in the stability of peroxisome proliferator-activated receptors as a result of activation by ligand

PPAR are transcription factors which are also subject to regulation by proteolytic degradation. Nonetheless, different PPAR are subject to different mechanisms of this regulation. Activating PPARα by ligand causes inhibition of ubiquitination, which leads to suppression of proteolytic degradation of this protein [80]. Ubiquitination of Lys^292^, Lys^310^, and Lys^388^ PPARα by muscle ring finger-(MuRF)1 results in export from the nucleus of this transcription factor and subsequently in proteolytic degradation of PPARα [81].

A similar mechanism occurs in regulation of the activity of PPARβ/δ by ligand. Activation by ligand causes inhibition of ubiquitination and inhibition of proteolytic degradation of PPARβ/δ [82].

Contrary to PPARα and PPARβ/δ, activation of PPARγ by ligand leads to ubiquitination and proteolytic degradation of this protein in proteasomes [83, 84]. In adipocytes, drosophila seven-in-absentia homolog 2 (Siah2) is responsible for this process [85]. Ubiquitination in this PPARγ mechanism causes changes in the structure of the activation function 2 (AF-2) domain, and as a consequence, the protein is directed to the proteolytic degradation pathway [86]. Nonetheless, PPARγ may also be subject to ubiquitination independently of ligand, which causes degradation or increase in the stability of the protein depending on the place of ubiquitination. Tripartite motif protein 23 (TRIM23) catalyzes polyubiquitination, owing to which PPARγ stability increases [87]. Meanwhile, F-box only protein 9 (FBXO9) [88] or makorin ring finger protein 1 (MKRN1) [89] has been identified as specific E3 ligase of PPARγ in adipocytes, which leads to ubiquitination and proteasome-dependent degradation of PPARγ. The regulation mechanism of PPARγ expression through proteolytic degradation is important in adipocyte differentiation, as this transcription factor impacts expression of genes responsible for lipogenesis of adipocytes. Meanwhile, MuRF2, which is present in cardiomiocytes, causes ubiquitination of PPARγ and thus leads to an increase in stability of the protein [90]. However, overactivity of MuRF2 causes polyubiquitination of PPARγ and, as a consequence, proteolytic degradation of the protein. It has been demonstrated that dysregulation of MuRF2 activity in diabetes has an impact on the development of cardiomyopathy [90].

## Phosphorylation changing the activity of peroxisome proliferator-activated receptors

Activated PPAR may also be subject to phosphorylation, which modifies the activity of these nuclear receptors. PPARγ is subject to phosphorylation into the N-terminus A/B domain by such kinases as protein kinase A (PKA) [91], extracellular signal-regulated kinase (ERK) MAPK [92–94] or JNK MAPK [92, 95]. This results in a change of character of the PPARγ. As a result of phosphorylation at the N-terminus, PPARγ ceases to be a transcription factor and begins to physically bind the activated NF-κB. This mechanism may constitute regulation of inflammatory responses in which activation of the above-mentioned kinases takes place and ligands for PPARγ are present.

PPARα is also subject to phosphorylation by such kinases as PKA [96], p38 MAPK [97] or ERK MAPK [98]. This causes enhanced ligand-dependent activation. Phosphorylation by p38 MAPK can also inhibit the activity of PPARα [99].

## Interaction between various peroxisome proliferator-activated receptors

All three PPAR have a similar LBD structure. This enables their simultaneous activation by the same ligand, for example, a given PUFA [33]. This results in interactions between different PPAR. As yet, unfortunately, not much research has been done into the interactions between various PPAR.

Activation of PPARγ leads to an increase in the expression of genes dependent on this transcription factor and on COX-2 in particular. This is associated with increased expression of PPARβ/δ [100]. However, sole activation of PPARβ/δ by ligand does not cause changes in COX-2 expression. It only cooperates with the activated PPARγ. Depending on the model, activation of PPARβ/δ inhibits [101] or increases [100] transcription activity of PPARγ. Activation of PPARβ/δ also increases expression of PPARα, whereas activation of PPARα decreases expression of PPARβ/δ [100]. Thus, simultaneous activation of PPARα and PPARγ leads to mutual abolishing of activity of both the PPAR through changes in expression of PPARβ/δ. However, the specific tissue expression of various PPAR should not be ignored, as it may modify the response to the activation of these nuclear receptors.

## Activation of peroxisome proliferator-activated receptor-γ as an inducer of cyclooxygenase-2 expression

Activating PPAR reduces inflammatory reactions. However, when pro-inflammatory factors are absent, activation of PPARγ induces the expression of COX-2 [100]. In the promoter of gene *PTGS2*, a PPAR response (PPRE) element is found [102, 103]. Thus, when pro-inflammatory factors are absent, activation of PPARγ causes induction of COX-2 protein expression. Inducers of COX-2 expression, which are commonly known, as compounds decrease expression of COX-2 in inflammatory reactions, are PUFA [γ-linolenic acid (γ-LA), AA, EPA, and DHA] [102, 104–106] as well as 15d-PGJ_2_ [107–109]. Nonsteroidal anti-inflammatory drug (NSAID) is also agonists for PPARγ [110, 111]. This is why such compounds as diclofenac, flufenamic acid, flurbiprofen, indomethacin, or NS-398, despite inhibiting the activity of COX-2, induce expression of that enzyme [107–109, 112].

AA and EPA (both PUFA) are processed into 2- and 3-series prostanoids [113–115], respectively. Prostanoids serve a very important biological function, unrelated to inflammatory reactions; they are crucial for proper functioning of the kidneys [116] and blood vessels [117]. Therefore, PUFA must influence the expression and activity of COX-2 differently than in inflammatory reactions. In metabolism, very often, the substrate of a given enzymatic pathway stimulates an increase in the activity of enzymes participating in its processing. That is a likely explanation as to why AA and EPA, by activating PPARγ increase the expression of COX-2. That is, they increase the expression and activity of the enzyme which uses them as substrates. This effect is not related to inflammation, but more likely to the functions of AA and EPA as substrates for the production of prostanoids.

## Peroxisome proliferator-activated receptor as a protein with anti-inflammatory properties: effect on NF-κB

Activation of all PPAR, PPARα [4, 118–121], PPARβ/δ [122, 123] and PPARγ [124–127] causes inhibition of NF-κB activation (Table 2). Nonetheless, the mechanism of anti-inflammatory properties is a very complex one and takes different forms.

**Table 2:** Presentation of mechanisms which involve inhibition of NF-κB activity by PPAR

PPAR isoform	The mechanism of the anti-inflammatory properties	Bibliography
PPARα, PPARβ/δ, PPARγ	Direct binding of p65 NF-κB	[30, 94, 128–130]
PPARγ	Activity of the E3 ubiquitin ligase, proteolytic degradation of p65 NF-κB	[131]
PPARβ/δ	Disruption in the assembly of the complex with TAK1, TAB1, and HSP27, disruption in the activation of NF-κB	[135–137]
PPARα, PPARγ	Binding of p300, inhibition of acetylation of p65 NF-κB	[30, 140, 141]
PPARα, PPARγ	Increase in the activity of SIRT1, decrease in acetylation of p65 NF-κB	[144–147, 150]
PPARα, PPARγ	Increase in expression of IκBα, inhibition of NF-κB activation	[154–156]
PPARβ/δ, PPARγ	Increase in expression and activity of PTEN, inhibition of NF-κB activation	[125, 157–161]
PPARα, PPARβ/δ, PPARγ	Increase in the expression and activity of HO-1, decrease in the level of ROS	[180–185]
PPARα, PPARγ	Increase in the expression and activity of SOD, decrease in the level of ROS	[71, 185, 197–200]
PPARα, PPARγ	Increase in the expression and activity of catalase, decrease in the level of ROS	[176, 177, 199, 204–206]

PPAR are transcription factors, but they also have properties which are not associated with the expression of genes. They are capable of binding different proteins, by which means they inactivate them. It is predominantly the binding of PPARα [30, 128], PPARβ/δ [129], or PPARγ with p65 NF-κB that is responsible for anti-inflammatory properties [94, 130] with p65 NF-κB which reduces the pro-inflammatory response.

The direct impact of PPARγ on NF-κB may be associated with its enzymatic properties. PPARγ is E3 ubiquitin ligase, which cooperates with E2 UBCH3. PPARγ causes ubiquitination of the Lys^48^ p65 NF-κB, which leads to proteolytic degradation of this NF-κB subunit [131]. The intensity of NF-κB degradation is increased by PPARγ ligand activation. However, it is not only NF-κB that is subject to such regulation by PPARγ. It is possible to reduce the stability of the MUC1-C by PPARγ, which has anticancer properties [132].

Inactivated PPARβ/δ occurs in the complex with p65 NF-κB [133, 134]. During induction of inflammatory responses, the inactivated PPARβ/δ is involved in activation of NF-κB p65. PPARβ/δ, in particular, takes part in assembly of the complex from TAK1, TAB1, and HSP27 [135]. Activation by ligand PPARβ/δ results in lack of this cooperation, and consequently, activation of PPARβ/δ interferes with the function of NF-κB p65. As a result, inflammatory responses caused by a high concentration of glucose, activation of the receptor for TNFα, IL-1β, or activation of TLR4 are reduced [136, 137].

PPARα and PPARγ can also inhibit acetylation of p65 NF-κB, which inhibits activation of this pro-inflammatory transcription factor. After degradation of the IκB, p65 NF-κB is subject to acetylation of the Lys^310^ p65 NF-κB by p300. This modification is very important with respect to the proper functioning of p65 NF-κB [138, 139]. PPARα [30, 140] and PPARγ [141] bind p300. Assembly of these complexes leads to loss of enzymatic properties of p300 and, as a consequence, to inhibition of activation of p65 NF-κB through reduced of acetylation of this NF-κB subunit.

Besides this pathway, PPARα and PPARγ also cause deacetylation of p65 NF-κB. The process of deacetylation, which leads to inactivation of NF-κB, is catalyzed by sirtuin 1 (SIRT1) [142–144]. Activation of PPARα increases expression and activity of SIRT1, which inhibits the p65 NF-κB function [144–147]. The impact of PPARα on SIRT1 is dependent on AMP-activated protein kinase (AMPK) [145, 146]. Activation of AMPK leads to phosphorylation of p300, which decreases activity of the latter enzyme [123]. Nevertheless, SIRT1 and AMPK are enzymes which activate one another [148, 149]. Hence, no accurate data are available on whether PPARα activates SIRT1 directly or activation of SIRT1 is caused directly by activation of AMPK. Activation of PPARγ causes deacetylation of p65 NF-κB depending on SIRT1 [150]. Nonetheless, the SIRT1 protein itself forms a complex with NCoR, SMRT, and PPARγ [149, 151, 152]. The process inactivates PPARγ.

PPARα and PPARγ indirectly impact the pro-inflammatory transcription factor. In particular, the promoter of the gene-coding IκBα is controlled by PPAR [153]. Owing to this, PPARα [154, 155] and PPARγ [156] increase expression of IκBα—protein binding NF-κB. As a result, inactive NF-κB bound with its inhibitor, IκBα, occurs in the cell. During inflammatory reactions, phosphorylation and proteolytic degradation of IκBα takes place, which activates NF-κB. Increased expression of IκBα by PPARα and PPARγ prevents activation of NF-κB.

Activation of PPARγ [125, 157–160] or PPARβ/δ [161] causes an increase in expression and activity of phosphatase and tensin homolog (PTEN). This effect, however, may be dependent on the research model. In, A549 line lung carcinoma, H23 line adenocarcinoma, and squamous H157 cell line carcinoma activation of PPARβ/δ decrease expression of PTEN for a few hours [162]. He et al. [163] shows that after a day of being exposed to ligand, expression returns to its control level. PTEN is phosphatase what catalyzes the dephosphorylation of phosphate from position 3′ in phosphatidylinositol-3,4,5-trisphosphate. This enzyme catalyzes a reaction reverse to PI3K. In the transmission of pro-inflammatory factor signals, activation of the PI3K/PKB/IKK/NF-κB pathway takes place [164]. Thus, increased expression and activity of PTEN leads to inhibition of NF-κB activation by PI3K.

## Peroxisome proliferator-activated receptor as protein with anti-inflammatory properties: effect on other signaling pathways

PPAR cause inhibition of inflammatory reactions not only by their effect on NF-κB. What is more, c-Jun is bound by PPARα or PPARγ. As a result of this reaction, inhibition of AP-1 activation and inhibition of AP-1 DNA-binding activity by activated PPARα occur [30, 50, 165], and PPARγ occurs [124, 141, 166]. AP-1-binding site occurs in promoters of many genes important in inflammatory reactions, including *PTGS2* [167]. As a result of this mechanism, PPARα and PPARγ inhibit COX-2 protein expression.

PPAR disrupt activation of STATs. In particular, PPARγ causes an increase in the expression of the suppressor of cytokine signaling 3 (SOCS3) [168, 169]. This protein inhibits activation of JAK2/STAT3. The activity of STAT1 is disrupted by the activated PPARα [170]. STAT5b is also disrupted by the activated PPARα or PPARγ [171, 172]. Nonetheless, the exact mechanism of such activity of PPARα and PPARγ is not fully understood. PPARα and PPARγ probably compete with STATs for coactivators [171]. It is also possible that they have an effect on membrane receptors. The expression of β-defensin 1 is increased, especially by PPARα. This signaling element leads to decreased expression of TLR4 in J774 macrophages [173].

## The effect on antioxidant enzymes

Besides the direct impact of the activated PPAR on NF-κB, an indirect effect on inflammatory reactions is also possible. PPAR reduce concentration of ROS by increasing the expression of antioxidant enzymes. Due to the fact that ROS fulfill a very important role as a second messenger in inflammatory reactions, the increase in the activity of antioxidant enzymes has an anti-inflammatory character [174, 175]. This leads to decreased activation of NF-κB, and thus to decreased expression of COX-2 [176, 177].

One of such ways is increased expression and activity of heme oxygenase-1 (HO-1) [2, 178]. In the HO-1 promoter, two PPAR responsive elements are present [179]. Owing to this, all the three isoforms of activated PPAR increase the expression of HO-1 [180–185]. An increase in HO-1 expression may also take place by other means. PPARγ forms a complex with Nrf2 and binds in antioxidant response element (ARE) on the HO-1 promoter, causing an increase in expression of this antioxidant enzyme [184]. PPARγ also leads to stabilization of mRNA HO-1, which prolongs the half-life of this transcript, thus increasing the expression of the HO-1 protein [186]. However, it must be remembered that activators of PPAR may also increase expression of HO-1 regardless of the transcription factor and depending on induction of oxidative stress [187].

HO-1 is an enzyme engaged in heme degradation to biliverdin and carbon monoxide (CO). These compounds have antioxidant properties. Biliverdin is converted to bilirubin, which is an antioxidant [188]. The second product of HO-1 activity, CO, decreases the activity of NADPH oxidase, which decreases the level of ROS in cells activated by pro-inflammatory factors [189, 190]. This also disrupts the activation of TLR4, which inhibits the pro-inflammatory effect of fatty acids and LPS [191]. In addition, CO, depending on ROS, causes S-glutathionylation of STAT3 and p65 NF-κB, which leads to deregulation of the function of these proteins [192, 193]. Owing to all of those properties, HO-1 is an antioxidant and anti-inflammatory enzyme [2, 194].

Activating PPAR causes induction of expression of other antioxidant enzymes, such as catalase, Mn-superoxide dismutase (SOD), and CuZn-SOD. In the promoter of gene Mn-SOD, a sequence of PPRE is present [195]. This allows PPARγ to increase the expression of this enzyme. The expression of CuZn-SOD is also increased after activation of PPARα [196–199] and PPARγ [185, 198, 200]. Consequently, an increase in SOD activity is followed by a decrease in O_2_^•−^ concentration, produced by NADPH oxidase.

In the promoter of the catalase gene, the PPRE is also present [201–203]. Owing to this, activation of PPARα or PPARγ causes an increase in expression of this antioxidant enzyme [176, 177, 199, 204–206]. As a consequence of the increase in the activity of catalase, the H_2_O_2_ level decreases and inflammatory reactions are reduced.

In this not fully understood mechanism, which is probably independent on ROS, an increase in catalase expression may lead to increased expression of COX-2 [207–209].

## Mechanisms inhibiting inflammatory reactions as the aim of the therapy

Inflammatory reactions, and the expression of COX-2 as well as synthesis of PGE_2_ in particular, constitute an important element in pathogenesis of many illnesses, such as Parkinson’s disease [210, 211], type II diabetes and concomitant diseases [212] and cancer [213, 214]. Therefore, the use of specific COX-2 inhibitors has a preventive effect and may facilitate the process of curing the diseases. Nonetheless, COX-2 is only an enzyme, whose expression and activity depends on intracellular signal transduction pathways induced in the course of many diseases. Therefore, the best solution in therapy is interference with the signaling pathways of the inflammatory reactions. In particular, PPAR activators can be used, such as naturally occurring *n*-3 PUFA (EPA and DHA), or artificial pharmacological compounds (ciprofibrate). Activating PPAR reduces inflammatory reactions and, as a result, decreases the expression and activity of COX-2. This allows for cancer prevention or for combining PPAR activators with the current anticancer treatment [215–224]. In addition to the effect on inflammatory reactions, medications that activate PPAR also regulate metabolism, which has a therapeutic effect in diabetics [225].

It should be remembered, however, that the activation of PPARγ may induce the expression of COX-2 and an increase in synthesis of PGE_2_ [102, 105, 107–112]. This may lead to counterproductive side effects of preventive treatment.

## Conclusion

Inflammatory reactions, such as all processes occur in living organisms, are strictly regulated. One group of such regulators are PPAR, receptors activated by nitrated fatty acid derivatives and cyclopentenone prostaglandins, products formed in the late stages of the inflammatory reaction. Activation of PPAR then results in the inhibition of core elements of the inflammatory reaction. Detailed knowledge of the regulatory mechanisms governing a given biological process facilitates interference with the functioning of cells and tissues, allowing for the development of therapeutic approaches for the treatment of diseases based on disorders in these processes.

## Abbreviations

5(*S*)-HETE: (*S*)-hydroxyeicosatetraenoic acid
15d-PGJ_2_: 15-Deoxy-Δ^12,14^-prostaglandin J_2_
AF1: Activation function 1
AMPK: AMP-activated protein kinase
ARE: Antioxidant response element
AA: Arachidonic acid
CO: Carbon monoxide
JNK: c-Jun N-terminal kinase
COX-2: Cyclooxygenase-2
CYP: Cytochromes P450
DHA: Docosahexaenoic acid
EP: Prostaglandin E_2_ receptors
EPA: Eicosapentaenoic acid
EET: Epoxyeicosatrienoic acid
ERK: Extracellular signal-regulated kinase
γ-LA: γ-Linolenic acid
HO-1: Heme oxygenase-1
H_2_O_2_: Hydrogen peroxide
IKKβ: IκB kinase β subunit
LBD: Ligand-binding domain
LOX: Lipoxygenases
MAPK: Mitogen-activated protein kinase
NO: Nitric oxide
NSAID: Nonsteroidal anti-inflammatory drug
NF-κB: Nuclear factor κB
NCoR: Nuclear receptor corepressor
PPAR: Peroxisome proliferator-activated receptors
PPRE: PPAR response element
ONOO^−^: Peroxynitrite
PTEN: Phosphatase and tensin homolog
PI3K: Phosphoinositide 3-kinase
PUFA: Polyunsaturated fatty acid
PG: Prostaglandin
PGE_2_: Prostaglandin E_2_
PKA: Protein kinase A
ROS: Reactive oxygen species
SMRT: Silencing mediator of retinoid and thyroid hormone receptors
SIRT1: Sirtuin 1
SOD: Superoxide dismutase
O_2_^•−^: Superoxide radical
